# Altering the availability of products within physical micro-environments: a conceptual framework

**DOI:** 10.1186/s12889-020-09052-2

**Published:** 2020-06-29

**Authors:** Rachel Pechey, Gareth J. Hollands, Patrice Carter, Theresa M. Marteau

**Affiliations:** 1 Behaviour and Health Research Unit, Institute of Public Health, University of Cambridge, CB2 0SR, Cambridge, UK; 2 Centre for Outcomes Research and Effectiveness, Research Department of Clinical, Educational and Health Psychology, University College London, WC1E 7HB, London, UK

**Keywords:** Availability, Intervention, Health-related behaviour, Conceptual framework

## Abstract

Altering the availability of products (e.g. food, alcohol or tobacco products) is one potential intervention to change behaviours to help reduce preventable premature deaths worldwide. However, research on these interventions lacks consistent conceptualisation, hindering clear reporting and cumulative synthesis. This paper proposes a conceptual framework – categorising intervention types and summarising constituent components – with which interventions can be reliably described and evidence synthesised. Three principal distinctions are proposed: interventions altering: (i) Absolute Availability (changing the overall number of options, while keeping the proportions comprised by any subsets of options constant); (ii) Relative Availability (changing the proportion comprised by a subset of options, yet keeping the overall number of options constant); (iii) Absolute and Relative Availability (changing both the overall number of options and the proportions comprised by subsets of options). These are subdivided into those targeting (a) a product or (b) a category of products. Mechanisms that might underlie each of these intervention types are discussed, and implications for future research highlighted. The proposed framework aims to facilitate study of a set of interventions that could contribute significantly to healthier behaviour across populations.

## Background

Non-communicable diseases are the leading cause of death worldwide [56]. Importantly, modifiable health behaviours, which include smoking, high alcohol intake, and excessive consumption of high calorie and nutrient poor foods [23], are major risk factors. These behaviours often occur in response to environmental cues, and are not necessarily driven by conscious decisions [48, 57]. Reflecting this, ‘nudge’ or ‘choice architecture’ interventions have recently been advocated as a means of changing population behaviour [49, 67, 79]. The specific focus of this paper is ‘choice architecture’ interventions that target the proximal physical micro-environment [35] – and in particular, interventions targeting Availability – i.e. altering the number of instances of a product within a physical micro-environment.

The choice architecture approach has theoretical foundations in dual process theories [29, 77]. As such, it has been hypothesised that interventions targeting choice architecture may provide a more effective way of changing predominantly automatic behaviour across populations than interventions primarily focused on providing information. Information-based interventions – when effective – can disproportionately benefit those who are least deprived [1, 51]. This is likely due to more deprived groups having fewer available cognitive and material resources – given cognitive resources may be depleted by both deprivation in childhood and current financial burdens [46, 54, 68] – as well as living in areas where there are more cues to less healthy behaviour [22, 27, 70], including greater availability of products such as energy dense foods, alcohol and tobacco. Interventions targeting the availability of less healthy products could be implemented at scale, in ways that rely less on limited cognitive resources than information-based interventions [33, 58].

There are many different types of behaviour change interventions, and restructuring the physical environment is one possible target – either standalone or as part of a multicomponent intervention – as identified in the Behaviour Change Technique (BCT) Taxonomy [52]. Breaking down the broader categorisations identified in the BCT Taxonomy, Hollands, Bignardi et al. [30] have developed a typology (TIPPME: Typology of Interventions in Proximal Physical Micro-Environments) of interventions in physical micro-environments, defined as those that “*alter the properties or the placement of objects or stimuli in proximal (sensorily perceptible) physical micro-environments*, *to elicit particular behaviours among people in those environments”.* The TIPPME typology classifies interventions into one of six different broad types, subdivided by their spatial focus, enabling more systematic design, reporting and evaluation of such interventions. These six types comprise availability (the focus of the current paper), position (e.g.*,* removing confectionery from end-of-aisle displays), functionality (e.g.*,* making a sugary drink package pour less freely), presentation (e.g.*,* removing or reducing branding), size (e.g.*,* making pack size smaller for higher energy snacks) and the information provided on products (e.g.*,* adding calorie labels on alcoholic drinks). TIPPME delineates physical environment interventions into specific categories according to intervention characteristics, in contrast to other approaches and frameworks, which outline steps for intervention development (e.g. Intervention Mapping) or take a broader assessment focused on intervention strategies or techniques [9, 38, 40]. In this paper, we aim to identify potential targets for intervening on Availability in physical micro-environments, and outline the corresponding possible mechanisms by which intervening on these factors might change behaviour. The focus of the current paper is therefore on developing a detailed conceptualisation of those interventions described in TIPPME as Availability interventions.

Interventions targeting Availability involve altering the number of instances of a product within the physical micro-environment (including, at the extreme, the absence of the product). It is important to note that this definition focuses upon a subset of environments, namely physical micro-environments, such as the interiors of shops, restaurants and bars, these being settings that people use for specific purposes and where they interact directly with objects and stimuli in those environments (i.e. excluding online supermarkets or similar) [30, 78]. This is in contrast to the broader physical macro-environments (e.g. infrastructure), a distinction drawn in the Analysis Grid for Environments Linked to Obesity (ANGELO) framework [78]. Within a small-scale environment such as a shop, the Availability of different products is likely to be directly observable to the customer when they are within that environment, whereas the Availability of a product across all the food stores in the area is unlikely to be known by an individual. A separate body of literature addresses the issue of Availability across a wider spatial area – e.g. focussing on the presence or absence of fruit and vegetables [2, 22]. Given the differences in scale, different mechanisms are likely to drive any effects, so these contexts should be considered separately. While the evidence for the impact of Availability within the physical micro-environment is still limited, recent reviews indicate the effectiveness of implementing Availability interventions in particular contexts, such as interventions that increase the proportion of healthier products in vending machines or workplaces [3, 25]. A recent Cochrane review of the impact of Availability interventions reports evidence – albeit limited by the quality and quantity of the included studies and therefore of low overall certainty – that such interventions can reduce selection and consumption of targeted food products – such as snack foods, higher energy main meal options or sugary drinks – in field settings such as schools, supermarkets and worksite cafeterias [31]. All the studies identified in this review related to altering the Availability of food products, with no studies identified for alcohol or tobacco products (an evidence gap that is beginning to be addressed [12]).

Given the promise of such interventions, greater attention to their design and reporting is merited, but we are unaware of any detailed conceptualisation of Availability interventions. This is reflected in the use of a range of terms in the literature on altering product Availability, with these applied inconsistently, leading to difficulties in conducting reliable evidence synthesis of intervention effects. See Table 1 for examples of how different studies have described altering the number of instances of product(s) within a physical micro-environment in a range of ways (for systematic reviews including Availability interventions, see [3, 13, 15, 25, 31]). Drawing on these systematic reviews reveals that while some studies have used the term Availability [8, 21, 60, 64, 83, 84], the terms Assortment Size, Assortment Structure, Food Provision and Stockpiling have also been used to describe conceptually similar interventions [4, 16, 72, 82]. Other more general terms such as “assortment”, as often used to describe the organisation of products in marketing research (see [17]), could relate to aspects of Availability – e.g.*.* the variety of products in a display – and/or an alternative set of interventions relating to products’ positioning, which is categorised separately to Availability in TIPPME [30].

**Table 1:** Example of terms used to describe interventions altering product Availability in physical micro-environments

Term used to describe intervention	Targeted change	Example reference(s)
Availability	Presence or absence of products	Wilcox et al. [84]: Including healthy options in choice sets in the laboratory Wilbur et al. [83]: Introducing lower-calorie options in vending machines
	Number of different products	Perry et al. [64]: Increasing the fruit and vegetable options in schools Bartholomew and Jowers [8]: Decreasing the number of less healthy entrées in schools
	Proportion comprised by a subset of products	Fiske et al. (2004): Increasing low-fat products and decreasing high-fat products in vending machines
Assortment size	Number of different products	Sela et al. [72]: Number of fruit and cookie options on university campus
Assortment structure^1^	Proportion comprised by a subset of products	Van Kleef et al. [82]: Increasing healthier products and decreasing less healthy products in a shop display
Food provision	Number of different products	Anderson et al. [4]: Increasing the fruit and vegetable options in schools
Stockpiling	Number of units of a particular product	Chandon & Wansink [16]: Units of particular brands of crackers, popcorn, fruit juice, noodles, oatmeal and granola bars in homes

^1^Van Kleef et al. refer to “assortment structure (i.e. availability)” in their abstract

Development of a coherent conceptual framework would facilitate cumulative understanding and synthesis of evidence, as well as provide a common language for identifying and discussing interventions across interested parties (researchers, policymakers, industry and the public).

The current paper has four aims:

I. to propose a framework for categorising Availability interventions.
II. to describe possible mechanisms underlying the effects of Availability interventions.
III. to outline how these mechanisms might relate to the proposed conceptual framework.
IV. to consider how the proposed framework could contribute to research in this area.

While we primarily focus on interventions targeting food products in this paper, the distinctions proposed should similarly apply to other product types.

## Conceptual framework

### Defining availability

In order to explore the mechanisms that could underlie interventions aiming to manipulate Availability, we need to define how we are using this term. In keeping with the recent Cochrane review of Availability interventions [31], we took the TIPPME [30] definition of Availability as our starting point: *“Add or remove (some or all) products or objects to increase, decrease, or alter their range, variety or number*”.

This definition encompasses a continuum from relatively small and commonly encountered changes, e.g. equivalent to fluctuating stock levels on supermarket shelves, to all units of certain products being removed, e.g. a ban on high-sugar drinks in hospitals. Table 1 sets out some examples of the ways in which Availability has been operationalised in the literature to date.

In this context, we could conceptualise Availability as: increasing or decreasing the number of units of a particular product; increasing or decreasing the number(s) of different products to alter the range, i.e. different brands, flavours and/or sizes of product(s); and/or altering the ratio of different subsets of products to alter the variety, i.e. types of product such as higher-energy vs. lower energy. Note that in this conceptualisation, range and variety overlap, and that altering one may also alter another. This is discussed further in the Framework section below.

### Proposed conceptual framework

**Fig. 1:**
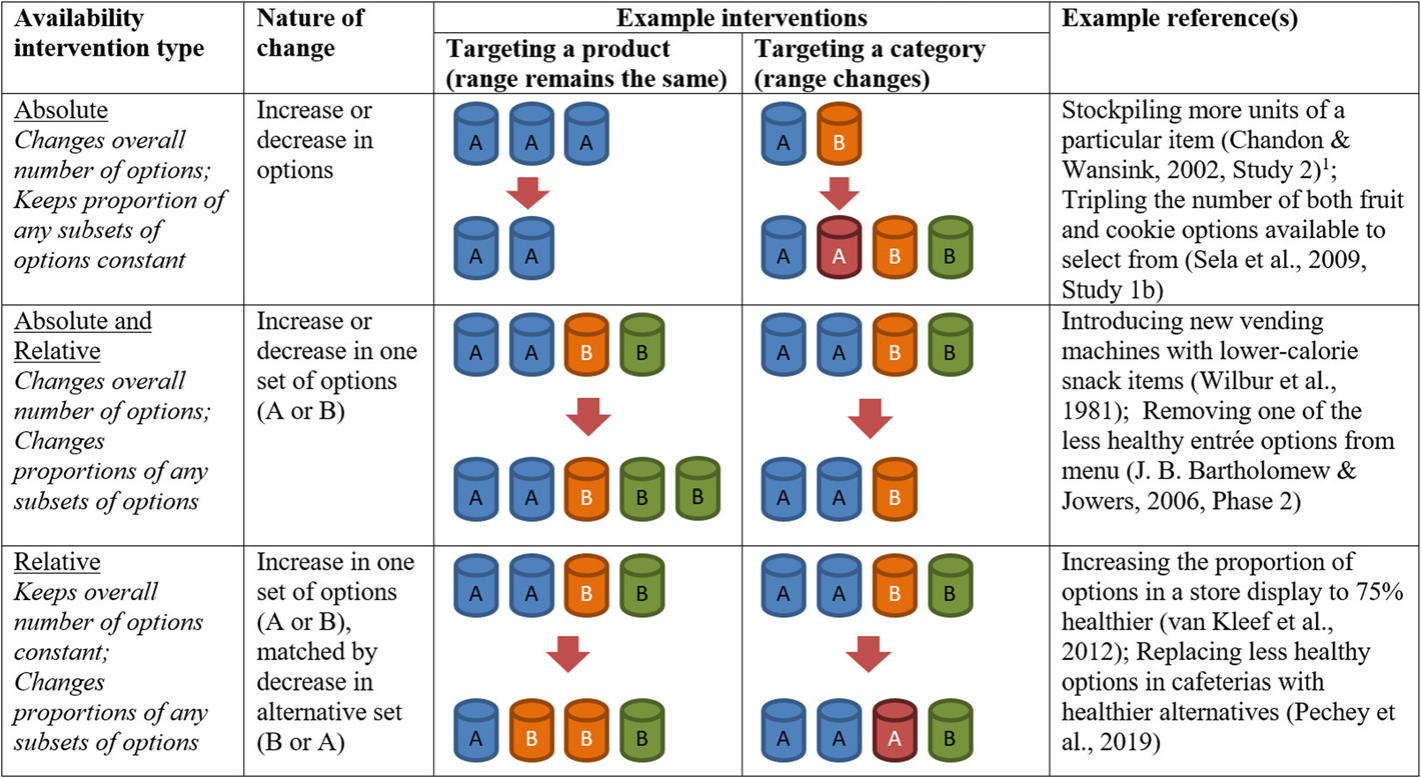
Proposed Availability intervention types. **A** and **B** represent different product sets (e.g. **A** could be healthier snacks and **B** less healthy snacks; or **A** larger chocolate bars and **B** smaller chocolate bars); colours are used to indicate different products within each product set. ^1^ N.**b**. In practice, this is likely to have unrecorded impact on Relative Availability, as other items are likely to be available but not assessed

At the most basic level, the nature of an Availability intervention involves changing (1) the number of target options, and sometimes also (2) the number of non-target options. As a result of these changes, the overall number of options and/or the proportion of target to non-target options may be altered. We propose interventions of Availability can be categorised into those (i) altering the overall number of options available (*Absolute Availability*), (ii) altering the proportion of a subset of options (e.g. lower energy foods) relative to other subsets (e.g. higher energy foods) (*Relative Availability*), or (iii) altering both Absolute and Relative Availability simultaneously (see Fig. 1). Regardless of whether studies set out to target the overall number of items vs. the proportion of a subset of items, this proposed categorisation focuses on the resultant changes following implementation. While this paper sets out examples primarily in the context of changing the food environment, these target options could be lower alcohol or alcohol-free drinks (rather than alcoholic drinks), or e-cigarettes (rather than cigarettes).

**Fig. 2:**
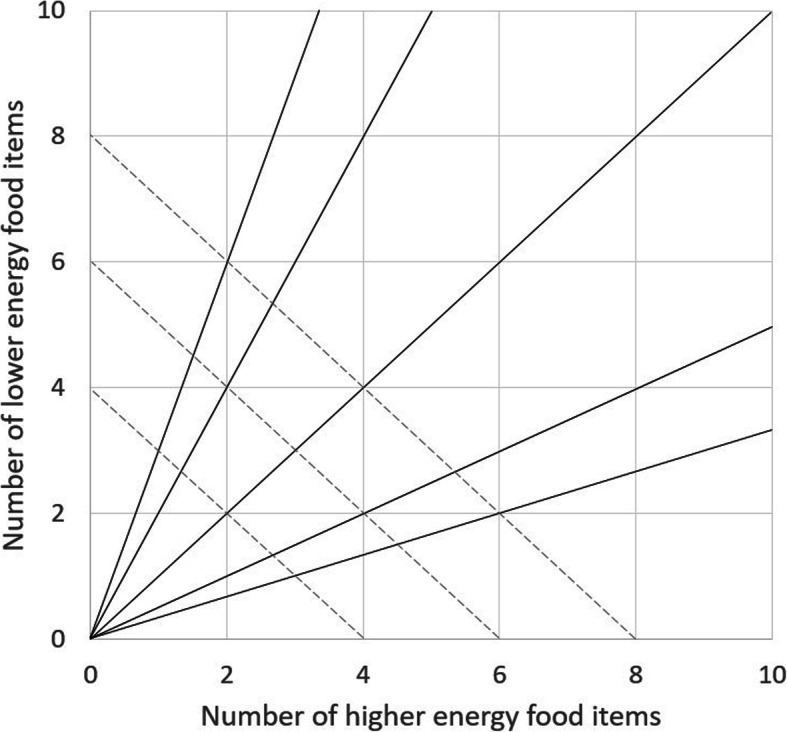
The space in which Availability can be intervened upon in the context of two subsets of products (lower energy and higher energy foods): Holding the number of lower energy items constant (horizontal lines); holding the number of higher energy items constant (vertical lines); holding the overall number of items available constant (examples shown as dashed rays); holding the ratio of lower energy: higher energy items constant (examples shown as solid rays)

Without consideration of this distinction between targeting the number of options and targeting the proportion of options, the targeted changes in Availability interventions may often lead to other inter-dependent changes in the number or ratio of items within that environment. For example, if a supermarket display contained two lower energy and two higher energy food items, and the number of lower energy food items was then increased from two to four, this would also change the number of available items (four to six) and the ratio of lower energy to higher energy items (1:1 to 2:1). Figure 2 illustrates this interdependency, showing different interventions that could be used in order to vary either the Absolute or Relative Availability of options, using the example of lower energy and/or higher energy foods. This shows how:

1. The Absolute Availability (number) of higher energy foods could be varied in the absence of lower energy foods (x axis);
2. The Absolute Availability (number) of lower energy foods could be varied in the absence of higher energy foods (y axis);
3. The Absolute Availability (number) of all foods could be varied (moving along one of the solid rays); and/or
4. The Relative Availability (ratio) of lower energy to higher energy foods could be varied (moving along one of the dashed rays).

In addition, given Availability can be changed by either altering absolute numbers of products and/or their relative proportions, it is important to note that comparing two equally spaced changes in the space shown in Fig. 2, may not lead to a similar change in behaviour. For example, the change from 1 to 3 lower energy items would likely lead to more substantial behaviour change than from 11 to 13 lower energy items (i.e. a non-linear relationship), as the proportional change is greater in the former scenario. This highlights the importance of reporting the baseline availability of options.

#### Target product vs. target category

In Fig. 1, we provide examples of Availability interventions in which the target is a product – i.e. the intervention alters the number of instances of an already available product and the range of products on offer stays the same – and another set in which the target is a product category – i.e. the product range is changed as new products are introduced or previously available products are removed. Although the basic Availability intervention could be the same in these two cases – e.g. increasing the number of options from two to three – the potential impact of interventions targeting options at these different levels of specificity could be very different. Take the example of decreasing the quantity of cans of a particular brand of soft drink on display from three to two – equivalent to what we might see if someone selected one of the cans from a store shelf – compared to decreasing the number of soft drink brands from three to two (equivalent to that brand no longer being on sale). If people make choices in line with their preferences, we might reasonably expect the latter to have a greater impact on selection of a soft drink, as people might find the removed brand more or less appealing than the others available. It would therefore be likely to change the relative appeal for each drink option more than reducing the quantity of a consistently available brand.

We have deliberately labelled the options in Fig. 1 as simply ‘A’ and ‘B’ as these could equally represent distinctions ranging from the general to the specific, such as less healthy foods vs. healthier foods, snack foods vs. main meals, chocolate bars vs. cereal bars, Brand X chocolate vs. Brand Y chocolate, or even different sizes of the same Brand X chocolate bar. We discuss this issue of specificity of option(s) targeted in the following section.

### Operationalisation

**Fig. 3:**
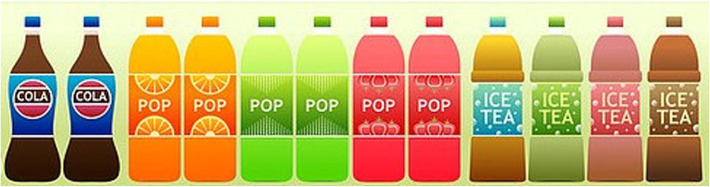
Example of a physical micro-environment where Availability could be intervened upon. Adapted from: https://pixabay.com/en/supermarket-shelf-products-snacks-1094815/

Availability interventions can be operationalised in terms of categories of interest (e.g. healthier drinks vs. less healthy drinks), the types of item available (e.g. cola), the brands available (“Brand X Cola”), or the units of a brand available (e.g. rows of Brand X Cola). This is illustrated in Fig. 3 below, where the available options can be counted at a number of different hierarchical levels:

1. Two options: carbonated soft drinks (Cola and Pop) or non-carbonated soft drinks (Ice Tea)
2. Three options: soft drink brands (Cola, Pop, Ice Tea)
3. Eight options: soft drink flavours (Cola, orange Pop, green Pop, red Pop, blue Ice Tea, green Ice Tea, red Ice Tea, brown Ice Tea)
4. Twelve options: rows of soft drinks (2 x Cola, 2 x orange Pop, 2 x green Pop, 2 x red Pop, 1x blue Ice Tea, 1 x green Ice Tea, 1 x red Ice Tea, 1 x brown Ice Tea)

Selecting any of these levels to group products when operationalising an Availability intervention will also impact on higher- or lower-levels of categorisation at the same time. For example, increasing the number of rows of Cola bottles will, by definition, increase the number of carbonated soft drink and Cola bottles. Conversely, increasing the number of carbonated soft drink options will require the researcher to decide whether to increase the number of Cola and/or Pop options or introduce new options. If the former, it also requires a decision on whether to increase the numbers of the flavours already available or introduce new flavours or new brands, and so on. Each of these decisions shapes the nature of the intervention, and potentially its impact, and thus necessitates careful recording of how the intervention is operationalised and the set of products altered.

Figure 3 also highlights that manipulating Availability may also impact on the positioning of products: for example, if red Pop bottles were removed and replaced with additional Cola options, those Cola bottles on the middle of the shelf may have a different impact on purchasing to those on the edge of the shelf, an effect which may occur in addition to any impact of increased availability.

## Potential mechanisms

**Fig. 4:**
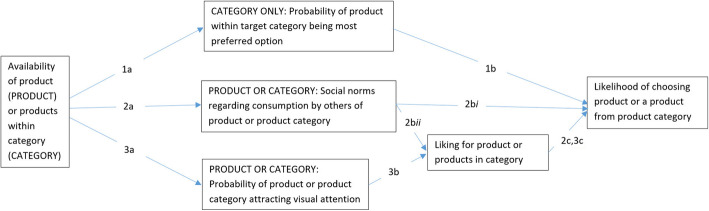
Potential (and not exhaustive) pathways from altering Availability to changing behaviour. 1. Availability → Probability of product in target category being most preferred option → Likelihood of selection. 2. Availability → Social norms altered to suggest greater or lesser consumption of these items by others → (Liking →) Likelihood of selection. 3. Availability → Probability of attracting visual attention → Liking (at least for liked items) → Likelihood of selection

In order to illustrate the value of developing a conceptual framework such as the one we have proposed above for Availability, we explore several mechanisms that could – singly or in various combinations and depending on context – underlie any impact of manipulating Availability on individuals’ behaviour. Figure 4 outlines five potential pathways that could be involved in impacting behaviour. These are not an exhaustive list of pathways, and it is important to note that the level of current evidence supporting the role of each pathway varies. We start by outlining the evidence for the key possible pathways in turn, and then consider how each might link to the types of Availability interventions proposed in the above conceptual framework.

### Proposed pathways

**1. Prior preferences.**

*Example: Adding apples to a display containing bananas and snack bars might increase the probability that a fruit option is customers’ most-preferred option, and therefore increase selections of fruit. In this example, if there were customers who would have preferred fruit, but do not like bananas, adding additional fruit options may lead them to swap their selection away from a snack bar.*

***1a. Increased availability → increased probability of product in target category being most preferred option***

Individuals may select items in accordance with their prior preferences for products within a set of available options. As items are added or removed, the relative ranking of the target product or category with regard to an individual’s preferences may change, changing the likelihood of each option being selected.

***1b. Increased probability of product in target category being most preferred option → increased likelihood of selecting target product***

This assumption underlies random utility theory in economics, whereby individuals chose the option that yields the greatest utility (i.e. the option that provides them with the greatest satisfaction or gain). As such, if an individual’s relative preference for a target option changes following a change in availability (as in 1a above), and this leads to the target option becoming the favoured option, or dropping from this position, this would then increase the likelihood of a change in behaviour.

**2. Social norms**

*Example: If a vending machine has few units of a particular type of product remaining, empty slots within the machine might imply greater popularity. The opposite pattern might be seen in cafeterias or supermarkets, where the greater presence of certain options –* e.g. *a greater number of types of chocolate bars* vs. *types of healthier snack bars – might imply greater popularity of these products in these contexts.*

***2a. Decreased availability [in the context of reduced quantity of stock] → Social norms updated to reflect greater consumption of target options by others***

***Increased availability [in the context of full stock] → Social norms updated to reflect greater consumption of target options by others***

Availability may alter social norms regarding consumption if individuals infer levels of consumption of target options by others when they observe the available number of products in specific contexts. This may subsequently impact on behaviour, given that when individuals have knowledge of others’ diet-related behaviour in the same setting, they are more likely to make similar selections or consume similar amounts [65, 66, 71].

Descriptive social norms – encompassing people’s beliefs about how common behaviours are in general or among individuals salient to them (e.g. people shopping in this supermarket are purchasing larger quantities of fruit and vegetables) – may be key. These are correlated with healthy eating behaviours [7], whereas injunctive norms – beliefs about what other people expect or approve of with regard to these behaviours (e.g. people should buy larger quantities of fruit and vegetables) – are not [43].

Studies manipulating social norm information often make others’ behaviour explicit – e.g., showing individuals lists that outline “previous participants’ selections”. Fewer studies have looked at the impact of implied popularity or implied social norms on behaviour. Importantly for their relevance to availability interventions, however, the influence of social norms does not have to involve seeing others. Behaviour can be changed through learning about the behaviour of others indirectly via environmental cues. For example individuals are more likely to select a healthier food option if they see empty wrappers suggesting that others have previously chosen this option [66].

It is unclear whether the opposite pattern might also be seen in cafeterias or supermarkets. For example, individuals may infer increased consumption by others if they observe greater numbers of target products available in contexts where the layouts have been deliberately determined and stock is (close to) full - for example, given awareness of the idea of supply (availability) and demand in commercial enterprises such as supermarkets. This may in turn lead to greater selection and/or consumption on the part of the individuals observing increased availability if this increase alters the perceived social norms regarding purchasing or consumption of these products.

***2b*i*. Social norms updated to reflect greater consumption of target options by others→ increased likelihood of selecting target option***

Deutsch and Gerard [19] proposed individuals follow social norms in order to: (a) enhance affiliation with social group – i.e. they want to be liked – and (b) to perform the ‘correct’ behaviour. Such modelling behaviour goes beyond mere imitation, involving an emotional component such as the desire to avoid social sanctions that may be imposed on those who do not follow such norms [10]. Higgs [28] proposes that following social norms is an adaptive behaviour, whereby following others in the context of diet makes people more likely to consume and share safe foods. For example, young children use social information to guide eating [74]. Given these possible motivations, updating of social norms regarding the selection or consumption of particular products by others could lead to changes in behaviour. Indeed, if social norms actually do achieve enduring behaviour change, then this could create a positive feedback loop via continual updating of social norms to reflect this changing behaviour.

***2b*ii*Social norms suggest greater consumption by others → increased liking for target option***

Updating social norms relating to the selection and consumption of a particular option might also impact on behaviour by altering the liking of an option. Providing social normative information has been shown to change liking for targeted foods and beverages [69]. This is reflected in reward-related brain activity [37, 59].

Another consideration is whether the influence of social norms may be moderated by food type and/or individual differences. For example, Pliner and Mann [65] found that social norms did not influence choices of unpalatable ‘healthy’ cookies over palatable ‘unhealthy’ cookies, while the results of Salmon et al. [71] suggest a social norm intervention was only effective at encouraging healthier choices if an individual had low self-control. It is possible that social norms may not be able to change liking sufficiently to influence behaviour when choosing between products for which there is a large existing discrepancy in liking.

***2c, 3c. Increased liking → increased likelihood of selecting target option***

Taste preferences and liking are reported to be among the most important influences on dietary behaviour [20, 24], including children’s food choices [14, 50]. Beyond self-report, manipulating implicit attitudes [26] – a measure of liking, which reflects the positive (vs. negative) associations of different food items to individuals – can result in altered food selection [32, 34], suggesting that emotions associated with the product can drive behaviour.

**3. Visual attention.**

*Example: If a display goes from containing 50% sugar-free beverages and 50% sugary beverages to 75% sugar-free and 25% sugary beverages, then there may be a greater likelihood that attention is drawn to the sugar-free beverages, given these take up a greater proportion of the visual field.*

***3a Increased availability → increased probability of target option attracting visual attention***

Increasing the availability of a target option relative to other non-target options would lead to the target options taking up greater space in the visual field. If an initial fixation point when first viewing a scene is random, then increased availability would increase the likelihood that a target option is the one initially observed. Evidence suggests, however, that initial fixation is likely to be on the most salient stimulus [36]. As such, the likelihood of initial fixation being on an option with increased availability would depend on its relative salience compared to the other options available. Following initial fixation, the attended-to location is transiently inhibited allowing attention to be redirected. When attention is redeployed, the increased proportion of the visual field dedicated to the option with increased availability may make this option more likely to attract attention. However, even with an increased visual presence due to increased availability, if individuals are actively searching for a particular option, they may not attend to these additionally available options, a phenomenon described as inattentional blindness [75].

***3b. Increased visual attention → increased liking for target option***

Increased visual attention may increase liking for a target option in line with the “mere exposure” effect [86]. This suggests that the appeal of a stimulus – such as Chinese ideographs, geometric shapes, or human faces – is increased after repeated image-based and physical exposures [18, 55, 85]. Moreover, gaze duration may both reflect and influence preference; in one study participants’ gazes began evenly distributed between two options, but then shifted to predominantly focus on the option they subsequently selected, whereas manipulating gaze duration biased decisions towards the more viewed option [73]. However, the mere exposure effect may require attention to be directed towards the stimulus [85], and consistency of context between exposure and testing [18]. In the context of research on food, the visual appeal (but not expected tastiness) of a food to children has been found to increase after viewing these items [11]. Therefore if increased availability draws visual attention to a set of options, this may in turn increase liking for these options – although the extent to which liking might extend beyond the visual domain is uncertain.

Evidence that exposure or increased visual attention can change behaviour is limited, and the handful of available studies provide equivocal evidence. Rangel and colleagues have conducted a series of laboratory studies looking at selecting between two or three food options, and developed models to predict selection [41, 42] (N.B. these models assume that initial gaze is random, contrary to possible influences of visual salience). They find increased visual attention increases the probability of selecting items self-reported as liked by participants, but decreases the probability of selection for disliked items [5].

In terms of field studies, the extent to which visual attention might be involved in manipulations is generally unclear. Some studies suggest increased sales when healthier options are placed at eye level [44, 80], but this positioning was also designed to increase the accessibility of these items, so the mechanism for an effect remains unclear. In contrast, Van Kleef et al. [82] found no effect of top vs. bottom shelf placement. Other studies suggest individuals prefer the middle option in an array [39, 53], perhaps because the centre of horizontal arrays receives more visual attention [6]. But these findings could potentially also be explained by ease of reach. As such, the potential for increased visual attention to lead to changes in behaviour in real-world contexts is yet to be demonstrated.

### Unintended changes

Unintended changes that may occur as a result of altering item availability include the physical proximity of products to individuals being simultaneously altered (e.g. lower-energy snack options may be placed at the front of a display, and therefore closer to individuals, when their number is increased). Altering the position of products in such a manner would be classified under a separate intervention type in the TIPPME typology [30]. While this would not be directly manipulated in Availability interventions, it is likely to vary (unsystematically) when Availability is altered.

## Linking mechanism and availability intervention type

In Section I above, we proposed three key types of availability intervention:

i. Absolute Availability
ii. Absolute & Relative Availability, and
iii. Relative Availability.

We further subdivided these into those targeting a product or a category.

For each of the mechanisms outlined in Section II above, we consider whether these might play a role in explaining any effects for the proposed categorisations of Availability interventions. To illustrate this, we explore these possible links between mechanisms and Availability type for the scenario of increasing the Availability of a target product or category.

For this discussion, we set aside the special case of the introduction of a product or category (i.e. going from zero to one or more instances of the product or category): Clearly, if a target product or category is not available, it cannot be selected. We also exclude altering Absolute Availability by targeting a product (i.e. altering the number of target products available when no other options are available), given this scenario is unlikely to be encountered in the real world.

*Pathway 1*

If the number of products in a target category are increased (and [for Relative Availability] the non-target options decreased), the target category may be more likely to include the most-preferred product amongst the available options (given the increase in target products *and/or* a decrease in non-target options that might remove a more desired product from the alternatives)

- *Applies to:*
  - *Absolute, Absolute & Relative, or Relative Availability*
  - *Target category only*

*Pathway 2*

If the number of (a) units of a target product or (b) products in a target category are increased relative to non-target options, it is more likely to increase perceived popularity of the target product/category (social norm) (in the context of relatively full stock levels)

- *Applies to:*
  - *Absolute & Relative, or Relative Availability*
  - *Target product or category*

*Pathway 3*

If the number of (a) units of a target product or (b) products in a target category are increased relative to non-target options, the target product/category is more likely to attract more visual attention (e.g. exposure effect)

- *Applies to:*
  - *Absolute & Relative, or Relative Availability*
  - *Target product or category*

*N.B. It is possible that visual attention would also change with Absolute Availability, if, for example, a particularly visually salient option was introduced, but this would vary with specific products and the visual context into which they are placed, rather than the extent of changes to target products or categories.*

It is also worth noting that we might expect differential effectiveness by Availability intervention type in certain contexts. Relative Availability, involving both increasing a target product and decreasing non-target products (or vice versa), would be expected to have a greater impact than Absolute & Relative Availability if the latter intervention only involved the equivalent increase in target products without the corresponding decrease in non-target options. By altering both target and non-target options, one would expect a greater likelihood that a more preferred option was present, greater visual attention to target options and greater updating to social norms. It is also plausible that in some contexts removing, e.g., less healthy options, may be more effective than adding healthier options, given differential liking for these each of these types of option – reflecting the findings in one online study targeting Availability [62].

## Implications for research

The current paper proposes a conceptual framework for Availability interventions, set out in Fig. 1, and maps this onto potential mechanisms underlying any effects. Adhering to a more systematic approach to conceptualising such interventions, facilitates: (1) an agreed terminology; (2) precision in study design and reporting and (3) explorations of mechanism. These in turn would allow for cumulative evidence synthesis, and the continued evolution of a shared language to discuss such interventions.

### Terminology

Table 1 showed examples of different terms that have been used to describe interventions that would be classed in this framework as Availability interventions. Some of the different terms used may reflect that Availability interventions can be manipulated in various ways, e.g. the presence vs. absence of a product; the number of different products; the proportion comprised by a subset of products. However, given each of these interventions involves the same key underlying change (altering the number of target (and perhaps also non-target) options available), use of a common terminology would aid researchers in identifying these conceptually linked studies.

In addition, our proposed framework allows us to understand and define how in some cases the reported target for change is not the only change. For example, Bartholomew and Jowers [8] targeted the number of entrees but this also altered the proportion of healthier to less healthy entrees resulting in an intervention that encompassed changes both to Absolute and Relative Availability. As such, agreeing a set of terms to describe different components of Availability may help to avoid such conceptual muddle.

### Study design and reporting

Different terms have been used to describe interventions that alter Availability, with little attention paid to the various ways in which these types of intervention can be implemented.

We suggest Availability interventions are disaggregated into the following components, albeit with the caveat that this information may not be available in all settings:

1. Aim
  - To alter: (i) Absolute Availability, (ii) Relative Availability or (iii) both
  - To target: product(s)/product categories
    *Absolute Availability: healthier cold drinks;*
    *Relative Availability: healthier vs. less healthy cold drinks*
2. Extent
  - The numbers of option(s) available in each option subset pre-intervention
  - The numbers of option(s) available in each option subset post-intervention
3. Operationalisation
  - The product(s) included in the assessment of the intervention, described at various levels of specificity if possible, *e.g. healthier cold drinks, of which there are four brands, three of which are diet soda and one juice*
  - How products are selected for removal or addition
  - The product range available in the physical micro-environment, *e.g. snack food and cold drinks in a vending machine*
4. Potential covariates
  - The extent to which the intervention of Availability also impacts on the positioning of products

See Supplementary Material for an example of information that would ideally be provided for an Availability intervention study.

Greater precision in study design enables a positive feedback loop through helping to identify the extent to which different components of Availability are contributing to any effects observed in studies. This in turn could identify the most effective intervention design, allowing the future studies to more precisely focus on the aspects of Availability that are most promising. For example, Steenhuis et al. [76] describe their Availability intervention as increasing the availability of low-fat products, fruits and vegetables within six product categories. However, this could potentially encompass adding one additional item or it could mean increasing the availability of such items ten-fold. Without further information on the nature of the intervention these results are uninformative. Even in better specified examples, applying the checklist suggested here reveals instances where details have not been clearly stated, e.g. an unspecified number of high-fat vs. low-fat entrees pre-intervention, for comparison with two high-fat and one low-fat post-intervention ([8]; Phase 1), making it harder to judge the extent to which this intervention altered the menu offering.

Moreover, without precise reporting of these details, synthesising results as if from equivalent interventions – e.g. combining across all identified Availability interventions – will obscure differences in effect from different types of Availability interventions, and could lead to invalid conclusions. Indeed, while we do not yet know if the differences proposed in the current paper would reveal differences in intervention effectiveness, consistent reporting will allow this to be systematically explored. For example, it is highly plausible that decreasing the proportion of less healthy entrées from two out of three options to one out of two (Absolute & Relative Availability ([8]; Phase 2), and increasing the low-fat options in vending machines from 5/28 to 8/28 (Relative Availability [21]), may be differentially effective interventions.

### Explorations of mechanism

Furthermore, a more systematic approach to conceptualising Availability interventions could lead to more nuanced explorations of mechanism by providing a focus on particular aspects of these interventions. (See Supplementary Material Table S1 for how some possible mechanisms may vary according to the proposed availability intervention types.) For example, by implementing an intervention altering the availability of a single product (i.e. keeping the range of products the same), studies could determine the magnitude of any effects of availability in the absence of any changes to relative preferences between the available options (i.e. excluding Pathway 1). Any such effects may instead involve for example, possible visual or social mechanisms. In contrast, by keeping the ratio of target to non-target items constant in studies targeting the Absolute Availability of products in a target category, these interventions are likely to be influenced more by the changed range of items than by increased visual attention or updating of social norms.

### Predicted impact on health inequalities

As an example of how examining the proposed intervention types in relation to their underlying mechanisms might help to progress research in this area, we consider briefly the possible impact of intervening on these different types of Availability on health inequalities. Given socioeconomic patterning in the healthiness of diets, with those poorest tending to have unhealthier diets particularly in relation to these containing less fruit and vegetables [45, 61], it is important that interventions targeting the availability of healthier foods do not differentially alter the food selections of those of higher socioeconomic status relative to those of lower socioeconomic status, thereby exacerbating existing inequalities. It is possible that the impact of Availability interventions on health inequalities may vary depending on the degree to which effects are driven by each of the potential mechanisms discussed above.

For example, if individuals’ selection of their most-preferred option was the predominant mechanism for the effects of Availability on behaviour, increased healthier food availability might widen health inequalities, given evidence of social patterning in food preferences [63, 81]. Such a scenario may be more likely if an intervention targets Absolute Availability.

Alternatively, if largely non-conscious processes (candidates being increased visual attention and/or salience) were substantially driving any impact of Availability we might expect intervention effectiveness regardless of prior preferences – suggesting more equitable impact of increased healthier food availability across socioeconomic groups. Evidence suggests that increased probability of attention being drawn to target options is likely to be a non-conscious process at least during initial fixation [47], but some evidence suggests later attention may also be drawn by more preferred options [73]. Further studies are needed to establish the extent to which these processes may be independent of existing preferences.

As such, impact on health inequalities could vary depending on the operationalisation of an Availability intervention – while as yet this is speculative, it calls attention to the need for a coherent evidence base that allows us to delineate mechanisms, which in turn could allow us to map these possible effects.

### Strengths and limitations

It is important to note that for this conceptual paper we did not set out to conduct a systematic review of the literature on Availability interventions knowing that we could draw on one already [31]. Instead, our delineation of Availability intervention types is drawn from describing different ways in which the number of target and non-target products can be simultaneously altered – the fundamental changes at the heart of altering Availability in physical micro-environments according to the TIPPME [30] definition: *“Add or remove (some or all) products or objects to increase, decrease, or alter their range, variety or number*”. We distinguish here between targeting a single product and targeting a product category, given the different potential mechanisms that could underlie such interventions. These categorisations may require further revision as the field develops. For example, we have limited the focus of this paper to interventions in physical micro-environments and to physical or spatial interventions. We have not included interventions that target temporal changes to Availability, such as changing the hours during which products are available for purchase, or the Availability of products over time. With these limitations in mind, we put forward this framework to provide a starting point from which further conceptualisation can develop.

Applying this framework has the potential to facilitate the synthesis of evidence, provide those reporting studies with guidance for improving the description of interventions, and raise questions for those designing interventions regarding the relative effectiveness of different possible intervention components. Moreover, we suggest that there may be different mechanisms underlying certain types of Availability interventions, or playing roles of different magnitude for others. Such predictions could be tested in future studies to help design and implement these types of interventions for maximum effect.

## Conclusions

This paper proposes the first conceptual framework for Availability interventions, a promising set of interventions for changing behaviour across populations to improve health for all. We consider potential mechanisms that might underlie any impact of Availability on behaviour, and illustrate how this might vary according to the proposed intervention types. Through examining the links between intervention types and mechanism, we highlight some potentially fruitful avenues by which this framework can contribute to research in this area.

Developing a framework to conceptualise and describe these types of intervention has the potential to strengthen the evidence base across this field of research, with possible benefits for tackling behaviours linked to obesity and other non-communicable diseases, and their resultant burden on global public health. While the extent of potential benefit is as yet unclear, if adopted, greater precision in design and reporting might shed considerable light onto the manner in which these promising interventions can be most effective and thereby provide the basis for optimising their impact.

## Supplementary information

**Additional file 1.** Example Study Information & **Table S1** [Availability intervention type by possible mechanism].

## Data Availability

N/A.

## Abbreviation

TIPPME: Typology of interventions in proximal physical micro-environments
