# Supplementary material for: Altering the availability of products within physical micro-environments: a conceptual framework
Source: BMC Public Health. 2020 Jun 29;20:986. doi: 10.1186/s12889-020-09052-2 (PMC7322874; doi:10.1186/s12889-020-09052-2)
Supplement: Supplementary file 1 — Additional file 1. Example Study Information & Table S1 [Availability intervention type by possible mechanism]. [file 12889_2020_9052_MOESM1_ESM.docx]

**SUPPLEMENTARY MATERIAL**

**Example Study Information**

| 1. Aim | - - - 1. To alter: (i) Absolute Availability, (ii) Relative Availability or (iii) both | Relative Availability |
| --- | --- | --- |
|  | - - - 1. To target: product(s)/product categories | Healthier (less than 5 grams sugar per 100ml) vs. less healthy (5 grams or more sugar per 100ml) cold drinks |
| 1. Extent | - 1. The numbers of option(s) available in each option subset pre-intervention | Healthier brands: 4 options  Less-healthy brands: 12 options |
|  | - 1. The numbers of option(s) available in each option subset post-intervention | Healthier brands: 8 options  Less-healthy brands: 8 options |
| 1. Operationalisation | - 1. The product(s) included in the assessment of the intervention, described at various levels of specificity if possible | Pre-intervention:  4 healthier brands, 2 of which are diet soda (available as both bottles and cans); 2 brands of water (bottles only)  12 less-healthy brands, 10 of which are soda (8 available as both bottles and cans; 2 just as cans), 2 brands of energy drinks (large cans only)  Post-intervention:  8 healthier brands, 6 of which are diet soda (available as both bottles and cans); 2 brands of water (bottles only)  8 less-healthy brands, 6 of which are soda (4 available as both bottles and cans; 2 just as cans), 2 brands of energy drinks (large cans only) |
|  | - 1. How products are selected for removal or addition | Removal: Brands with highest sugar grams/100ml removed  Addition: Drinks matched to those removed by flavour (e.g. cola) and pack size (e.g. 330ml can) as far as possible |
|  | - 1. The product range available in the physical micro-environment | Single-serve bottles and cans of cold drinks, including sodas and bottled water, in hospital vending machines |
| 1. Potential covariates | The extent to which the intervention of Availability also impacts on the positioning of products | The new products are placed in the position in which the product they were matched to replace was previously. The same number of units are put in the vending machine for each product (i.e. brands have a row for each pack size; rows are filled to capacity). |

**Table S1**. Availability intervention type by possible mechanism

| **Availability intervention type** | **Example interventions** | | **Possible mechanisms beyond selection of most-preferred option** |
| --- | --- | --- | --- |
|  | **Targeting a product (range remains the same)** | **Targeting a category (range changes)** |  |
| Absolute  *Changes overall number of options; Keeps proportion of any subsets of options constant* | A  A  A      A  A | B  A  B  B  A  A | *Target category:*  Consider possible positioning effects |
| Absolute and Relative  *Changes overall number of options; Changes proportions of any subsets of options* | B  B  A  A  B  B  B  A  A | A  A  B  B  B  A  A | *Target product/ target category:*  Social norms;  Visual attention;  Consider possible positioning effects |
| Relative  *Keeps overall number of options constant;*  *Changes proportions of any subsets of options* | B  A  B  B  B  B  A  A | A  B  A  A  A  A  B  B | *Target product/ target category:*  Social norms;  Visual attention;  Consider possible positioning effects |
| *Does most-preferred option explanation apply?* | No: Range of options remains constant | Yes: Effects could be due to people selecting their most preferred option |  |
